# Pettigrew's Medical Portrait Gallery

**Published:** 1839-01

**Authors:** 


ART. IV.—*The Medical Portrait Gallery.* Nos. IV.—IX. By T. J. PETTIGREW, F.R.S. &c.—*London,* 1838.

SINCE we noticed this work in our eleventh Number, six more Parts have been published, containing portraits and memoirs of the following distinguished persons: Dr. Blundell, Caius, Morgagni, Radcliffe, Bichat, Sir A. Cooper, Dr. Copland, Dr. Cooke, Mead, Dr. William Hunter, Dr. Jenner, Dr. Baron, Dr. Baillie, Dr. Bright, Sir B. Brodie, John Hunter, Mr. Lawrence. We are much pleased to find that Mr. Pettigrew has obtained sufficient encouragement to induce him to proceed with the publication; as, with some considerable faults of plan and execution, it is unquestionably a very interesting and attractive work, and, we trust, will obtain the general patronage of the medical profession. The portraits are, in general, remarkably good, both as to the likeness and execution; and the whole publication is extremely elegant and singularly cheap. The more recent parts contain a much larger proportion of the living members of the profession than the earlier ones; but we trust the author intends to give us all the really illustrious men of former days, of whom authentic portraits exist, before he descends from the higher class of his contemporaries, to cater to the vanity of those who, however estimable in their own circle and in their own generation, possess not that buoyancy of genius and knowledge which will enable them "to tread the waves of glory" through the ages that come after ours. Hitherto, with one or two exceptions, Mr. Pettigrew has avoided this open snare; and we name it now more as a friendly warning for the future than in reproof of the past. We renew our warning also against the superfluous glorification of the living; although, as we can hardly believe that these biographies of his contemporaries are drawn up without the knowledge of the subjects of them, we shall henceforth be disposed to reserve our censures (if there be cause for such) rather for the vanity of the painted than the unworthy adulation of the painter. Of the proper subjects of biography, the dead, let the truth be boldly spoken, whether good or evil; but, as it is impossible to censure a man who, if he is not our personal friend, has, at least, shown us courtesy by sitting to us for his portrait, or has supplied us with the materials for its composition, so we ought, in fairness, to restrain ourselves from all unseemly praise, and assuredly from all flattery. Without any thing of this kind, enough remains to the contemporary biographer, in the statement of undoubted *facts*,—which, after all, are, to the worthy, the truest praise. Of this kind are the exact age, place of birth, studies, nature and date of appointments, publication of writings, &c. &c.; and we are sorry to say that, in such useful information as this, some of the biographical notices are very deficient. We particularly request Mr. Pettigrew's attention to the subject last mentioned. It would be an easy task for him to make each of his notices accurately *bibliographical* as well as *biographical;* and if he could assure his readers that they might refer with confidence to his work for authentic records of the title, date, and extent of all the writings of the individuals commemorated, as well as of the more prominent points in their personal history, he might rest assured that his work would not only deserve but obtain that permanent station in literature which ought to be the aim of every author who is actuated by worthy motives. If these things were attended to, we could well excuse the omission of Mr. Pettigrew's criticisms on writings with which most of his readers are familiar; and still better could we spare the heavy truisms and the threadbare poetical quotations with which it is the author's pleasure to load his pages. The general character, indeed, of the memoirs is that flat mediocrity which used to be endurable neither to gods, to men, nor to columns; and which is assuredly more distasteful to reviewers and critics than the most glaring faults that, in works of another class, are relieved by the thousand attendant compensations of genius. A friend, to whom we showed these memoirs, and whose opinion of them we requested, gave it in the words of the satirist:

> "Too bad for a blessing, too good for a curse,
> I wish from my soul they were better or worse."

We hope Mr. Pettigrew will take care so to improve and perfect his work as to put it out of the power of any one, who like himself may have a passion for mottoes, justly to adopt these lines as the general epigraph of his biographies.

